# Characterizing the Different Effects of Zika Virus Infection in Placenta and Microglia Cells

**DOI:** 10.3390/v10110649

**Published:** 2018-11-18

**Authors:** Maria del Pilar Martinez Viedma, Brett E. Pickett

**Affiliations:** 1 J. Craig Venter Institute, 4120 Capricorn Lane, La Jolla, CA 92037, USA; pviedma@jcvi.org; 2 J. Craig Venter Institute, 9605 Medical Center Drive, Rockville, MD 20850, USA

**Keywords:** zika virus, placenta cells, microglia cells, siRNA, TLR7/8

## Abstract

Zika virus (ZIKV) is a neuropathic virus that causes serious neurological abnormalities such as Guillain-Barre syndrome in adults and congenital Zika syndrome (CZS) in fetuses, which makes it an important concern for global human health. A catalogue of cells that support ZIKV replication, pathogenesis, and/or the persistence of the virus still remains unknown. Here, we studied the behavior of the virus in human placenta (JEG-3) and human microglia (HMC3) cell lines in order to better understand how different host tissues respond during infection. We quantified the host transcriptional response to ZIKV infection in both types of cells at 24 and 72 h post-infection. A panel of 84 genes that are involved in the innate or adaptive immune responses was used to quantify differential expression in both cell lines. HMC3 cells showed a unique set of significant differentially expressed genes (DEGs) compared with JEG-3 cells at both time points. Subsequent analysis of these data using modern pathway analysis methods revealed that the TLR7/8 pathway was strongly inhibited in HMC3 cells, while it was activated in JEG-3 cells during virus infection. The disruption of these pathways was subsequently confirmed with specific small interfering RNA (siRNA) experiments that characterize their role in the viral life cycle, and may partially explain why ZIKV infection in placental tissue contributes to extreme neurological problems in a developing fetus.

## 1. Introduction

Zika virus (ZIKV) is an enveloped positive-sense, single-stranded RNA virus that belongs to the *Flavivirus* genus of the *Flaviviridae* family. Viruses belonging to this taxon include several human pathogens, such as yellow fever (YFV), dengue (DENV), Japanese encephalitis (JEV), tick-borne encephalitis (TBEV), and West Nile (WNV) viruses. ZIKV is transmitted mainly by *Aedes* spp. mosquitoes, and was originally discovered in 1947 in the blood of a febrile Rhesus monkey in Uganda’s Zika forest [1].

Most ZIKV infections were associated with mild symptoms characterized by fever, rash, joint pain, and conjunctivitis. However, the recent worldwide epidemic has demonstrated that ZIKV can exhibit neurotropism that causes serious neurological abnormalities in humans. Specifically, Guillain-Barre syndrome has been observed in adults after infection, and congenital Zika syndrome (CZS) has been observed in the fetuses of infected mothers, with microcephaly being one of the most devastating consequences of this infection [2,3]. After a surge in microcephaly cases was associated with the recent severe Zika virus outbreak in Brazil [4], the World Health Organization declared ZIKV to be a Public Health Emergency of International Concern on 1 February 2016.

A marked difference between Zika and other flaviviruses is that ZIKV can be sexually transmitted [5,6,7,8] and is part of the “TORCH” pathogens, which include *Toxoplasma gondii*, *Listeria monocytogenes*, human immunodeficiency virus (HIV), varicella zoster virus, and other infectious agents that are associated with congenital anomalies [9]. The most recent studies have demonstrated that ZIKV is highly neurotropic; it infects and crosses the placenta in pregnant women, can be vertically transmitted from the infected mother to the fetus, and can produce CZS [10].

Despite a large effort by researchers, the molecular mechanism(s) of the vertical transmission of infection across the placenta barrier is still unknown [10]. Various cell types in the placenta are susceptible to infection with Zika virus [11]. Specifically, observations of hydropic and hyperplastic chorionic villi, as well as the proliferation of Hofbauer cells, have been reported during infection with Zika virus [12]. However, these cellular effects have not been shown to significantly impact placental function [12,13]. It is difficult to establish accurate laboratory models, since there are substantial anatomical differences between the placenta in humans versus other animals. Although human cell lines cannot currently replicate all of the gestational stages or the inherent structure of the human placenta, they are a useful initial tool to better understand the mechanism of action of teratogenic pathogens. Recognizing both the practicality and the limitations of these cell lines will help researchers better interpret experimental results.

Recent studies have described the cellular innate immune response against ZIKV infection, specifically, the role of Toll-like receptors [14,15], as well as specific molecules involved in this response such as STAT2 [16,17]. In this work, we better characterize the role of the intracellular immune response during Zika virus infection. To do so, we infected either human placenta or human microglia cell lines with ZIKV, and studied their innate immune response against ZIKV infection across multiple time points. Specifically, we studied the role of TLR7, TLR8, and STAT2 during ZIKV infection, since they are key molecules in the cellular antiviral and IFN-mediated responses. This work improves our understanding of how the virus is able to cross the placental barrier and the role of microglia cells during infection of the fetal brain. We demonstrate that the intracellular response of specific immunological pathways against ZIKV infection differs depending on the type of cells being infected.

## 2. Materials and Methods

### 2.1. Cells and Virus

Microglia HMC3 (CRL-3344), epithelial placenta JEG-3 (HTB-36), and epithelial Vero (CCL-81) cells were purchased from the American Type Culture Collection (ATCC, Bethesda, MD, USA) and maintained in high-glucose Dulbecco’s modified Eagle’s medium (DMEM), containing 10% fetal bovine serum (FBS; HyClone Laboratories, South Logan, UT, USA) and 1% penicillin/streptomycin at 37 °C in a 5% CO_2_ incubator. ZIKV Puerto Rican strain (PRVABC59) was obtained through Biodefense and Emerging Infections (BEI) research resources (Zika Virus, PRVABC59, NR-50240).

### 2.2. Cell Infection

Cells were plated one day prior to infection in complete DMEM at 37 °C in a 5% CO_2_ incubation chamber. ZIKV stock was diluted in DMEM with 2% FBS to obtain an inoculum with a multiplicity of infection (MOI) of 0.01, which was then incubated with the cells at 37 °C in a 5% CO_2_ incubation chamber for one hour and rocked every 15 min. The inoculum was removed after one hour, complete DMEM was added, and cells were incubated at 37 °C in a 5% CO_2_ incubation chamber until the collection time point.

### 2.3. LIVE/DEAD Cellular Assay

The cytopathic effects (CPE) of ZIKV infection on the different cells was measured by using the LIVE/DEAD Cell Imaging Kit (ThermoFisher, R37601, Waltham, MA, USA), based on a cell-permeable dye for staining live cells and a cell-impermeable dye for staining dead and dying cells, which are characterized by compromised cell membranes. For this assay, cells were plated and ZIKV-infected at a MOI of 0.01 and incubated for five days. Supernatants were collected at each time point and saved for viral titering. In parallel, cells were exposed to the staining reagent included in the LIVE/DEAD Cell Imaging Kit for 15 min. The image data were captured using a Celigo Imaging Cytometer (Nexcelom Bioscience, Lawrence, MA, USA) at different times of infection, identifying healthy cells in green and damaged cells in red.

### 2.4. RNA Extraction, cDNA, RT-qPCR, qPCR Arrays, and Standard Curve

RNA from mock or ZIKV-infected cells was extracted from samples using the RNeasy mini kit from Qiagen (Hilden, Germany). Subsequent cDNA synthesis was performed with the SuperScript™ III First-Strand Synthesis SuperMix (ThermoFisher 18080400, Waltham, MA, USA). RT-qPCR was performed using specific primers for gene expression experiments (IDT; Supplementary Table S1) and PowerUp™ SYBR^®^ Green Master Mix (ThermoFisher A25778, Waltham, MA, USA). The RT^2^ Profiler PCR Array Human Innate and Adaptive Immune Responses (Qiagen, Hilden, Germany) was used following the manufacturer’s protocol (Supplementary Table S2) for pathway-focused gene expression analysis.

The ZIKV standard curve was established using custom TaqMan Primers and Probe (IDT; Supplementary Table S3). Briefly, 10-fold serial dilutions of ZIKV genetic material from BEI resources (NR-50244) were used to establish the correlation between the cycle threshold (Ct) value and the number of molecules/µL of viral RNA. The RNA copy number (number of molecules/µL) was calculated as: [RNA concentration (g/mL)]/[RNA transcript length (nucleotides) × molecular weight of a nucleotide (330 g/mol) × Avogadro’s number (6.023 × 10^23^). In the same way, RNA from ZIKV stock (BEI, NR-50240) was serially diluted to establish the correlation between Ct and PFU/mL.

Separate primers and probes were designed for the Asian and African lineages, with the African lineage used as a negative control in these sets of experiments. Specifically, bioinformatics analysis was performed to identify the best sequences for primers and probes. This workflow consisted of: (1) collecting available Asian and African ZIKV complete genomes from the Virus Pathogen Resource (ViPR) database [18], (2) generating a multiple sequence alignment with MAFFT [19], (3) performing a statistical analysis to identify nucleotide positions that significantly differed between Asian and African lineages [20], and (4) confirming the specificity of the reagents using BLAST [21].

### 2.5. Plaque Assays

Viral titers were determined by plaque assay on Vero cells, which were plated one day before the assay at 90% of confluency in complete DMEM at 37 °C in a 5% CO_2_ incubation chamber. Supernatants collected at various time points after infection were serially diluted and inoculated on the cells for one hour, with rocking every 15 min, at 37 °C in a 5% CO_2_ incubation chamber. After that, a 1:1 mixture of 0.6% agarose and DMEM with 2% FBS was added and incubated at 37 °C in a 5% CO_2_ incubation chamber. The overlay was removed after three days, cells were washed with phosphate-buffered saline (PBS), and stained and fixed with a crystal violet solution (2 g crystal violet + 60 mL 100% Ethanol + 40 mL 37% Formaldehyde + 100 mL PBS) for 30 min at room temperature in the dark. The excess crystal violet solution was removed by washing with water. After the plates were dry, plaques were visualized, and the number of plaque-forming units (PFU)/mL was determined. All of the experiments were performed in triplicate.

### 2.6. siRNA Experiments

ON-TARGET plus SMARTpool siRNA for human TLR7, TLR8, and STAT2 together with positive and negative controls were purchased from Dharmacon (51284, 51311,6773, D-001830-10-05, D-001810-10-05 respectively) and transfected into the cells with the transfection reagent Lipofectamine™ RNAiMAX (ThermoFisher 13778030, Waltham, MA, USA) following the manufacturer’s protocol. Cells were infected with virus eight hours after transfection, once the effect of the siRNA was corroborated. Efficiency controls for the siRNA experiments were included (Supplementary Figure S1), and were used as the negative control to normalize the gene expression data. Supernatants from cell cultures were collected for viral titering at one day or three days post-transfection in each cell type, and at one day or three days post-transfection and subsequent viral infection in each cell type. Cells were washed with PBS and collected at the same time points for RNA extraction.

### 2.7. Signaling Pathway Analysis

The R implementation of the signaling pathway impact analysis algorithm (SPIA) was used to predict activated or inhibited pathways. Specifically, pathway data from five publicly available databases were used to generate a null distribution of the genes in each signal cascade pathway (1000 bootstrap replicates) and calculate a Bonferroni-corrected *p*-value in order to account for multiple hypothesis testing. The total net accumulated perturbation in each pathway was calculated by using the differential expression values and pathway topology. Positive perturbation values indicate pathway activation, while negative values represent pathway inhibition. Pathways that contained a statistically significant number of detected differentially expressed genes in the different cell types at each time point were categorized as either “activated” or “inhibited” in the algorithm output and subsequently ranked by *p*-value [22].

### 2.8. Statistical Analysis

Data analysis was performed using Prism (GraphPad Software, La Jolla, CA, USA) for statistical analysis. All of the experiments were carried out in triplicate, and values were presented as means ± SEM. *p*-values of these experiments were calculated with a non-paired Student’s *t*-test. Statistical significance was accepted at *p* < 0.05.

## 3. Results

### 3.1. ZIKV Production, Titer, Cell Infection, and Cytopathic Effects (CPE)

**Figure 1:**
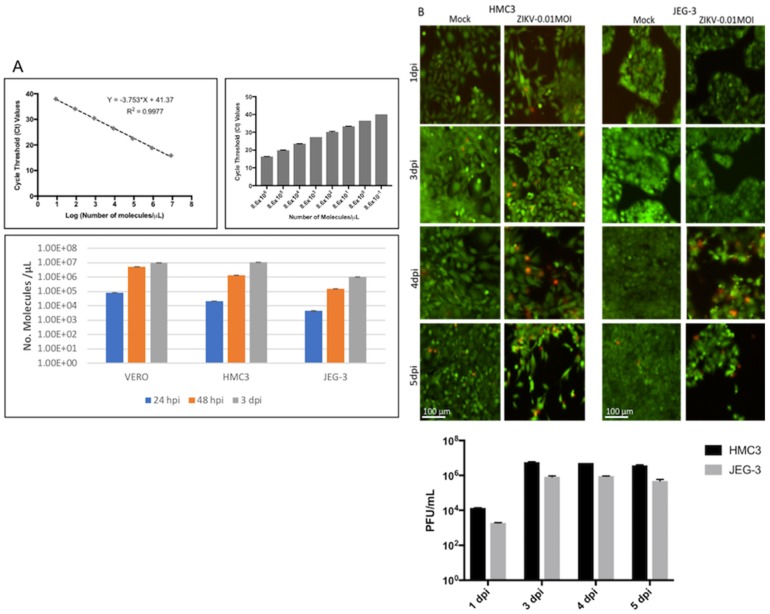
Quantification of Zika virus (ZIKV) titer, replication, and cytopathic effects (CPE) over time. (**A**) RT-qPCR standard curve to measure the number of virus genomes (upper panels); RT-qPCR to quantify ZIKV molecules in VERO, HMC3 and JEG-3 cells after one, two, and three days of infection. (**B**) CPE induced by ZIKV infection in HMC3 and JEG-3 cells at one, three, four, and five days post-infection, with the green stain representing healthy cells and the red stain indicating unhealthy and/or dying cells and the scale bar representing 100 micrometers. The lower panel shows the results from plaque assays performed in triplicate to quantify the ZIKV particles released to the media at each time point. Error bars represent standard deviation.

After infecting different cell lines with ZIKV at 0.01 MOI, we showed that ZIKV was able to replicate in both cell lines (HMC3, JEG-3) as well as in the control cells (VERO), increasing its replication along the time of infection (Figure 1A). A standard curve was generated to establish the correlation between Ct values and the number of molecules/µL of viral RNA (Figure 1A upper panel) using a ZIKV-specific TaqMan probe. It is of note that the rate of ZIKV replication was at least 10-fold lower in placenta cells than in microglia or VERO cells when quantified by qPCR (Figure 1A lower panel) as well as through plaque assays (Figure 1B, lower panel). When we performed a live/dead assay, we observed CPE in ZIKV-infected placenta and microglia cells starting to appear at four days post-infection. Compared with the mock-infected cells, the images captured immediately after the assay showed a steady number of healthy cells (green) over time in mock-infected samples, and a decrease in the number of cells in ZIKV-infected samples (Figure 1B). This decrease is due to the increased detachment and loss of dead cells, as well as an increase in damaged cells (red) (Figure 1B, upper panel). In order to quantify the levels of infection in each time point, we performed plaque assays to determine the viral particles released to the media along the time (Figure 1B, lower panel). Based on these initial results, we chose one day post-infection (dpi) and three dpi as the optimal time points for further experiments, with the aim of studying the kinetics of the intracellular transcriptional response during viral infection before transcription associated with the cytopathic effects overcomes the virus-specific transcriptional signal.

### 3.2. Host Intracellular Innate Immune Response and Differentially Affected Signaling Pathways

**Figure 2:**
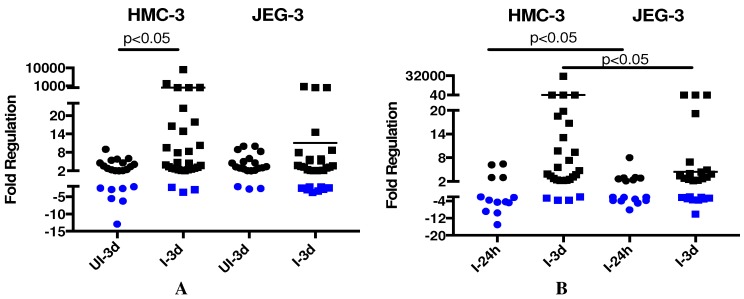
Results from an intracellular innate immune response RT-qPCR array. Each circle or square represents an individual gene that was either upregulated (black) or downregulated (blue). (**A**) Differentially expressed genes at one dpi versus three dpi in human microglia and placenta cells. (**B**) Differentially expressed genes of ZIKV-infected (I) samples versus time-matched mock-infected (UI) samples at 24 h and three days post infection with ZIKV. The numerical values on the Y-axis have been collapsed in regions that had no differentially expressed genes in order to improve visibility.

ZIKV is able to cross the placenta in pregnant women and infect the fetal brain; however, the mechanism by which this occurs has not been fully elucidated. To better understand the intracellular transcriptional response in placenta and microglia cell lines during ZIKV infection, we used RT-qPCR to evaluate an unbiased panel of 84 genes involved in the human innate and adaptive immune response (Supplementary Table S2). The results of this analysis are shown in Figure 2, where each dot or square represents a single transcript. When we compared the differentially expressed transcripts at one dpi versus three dpi (Figure 2A), there was a significant difference in the transcriptional regulation in infected cells compared with uninfected cells in microglia cells, but there was not a difference in placenta cells. Also, when we checked infected (I) cells versus uninfected (UI), a significant difference in differentially expressed genes from microglia cells compared with placenta cells at both time points one dpi and three dpi was observed (Figure 2B).

The significant differences observed in the RT-qPCR data led us to hypothesize that genes in specific signaling pathways were differently affected in each cell type within three days after ZIKV infection. Therefore, we analyzed these data using the signaling pathway impact analysis algorithm (SPIA) to identify the signaling pathways (i.e., networks of genes flowing from receptors to transcription factors) that were either significantly activated or inhibited in the different cells and at the different time points (Table 1). The summarized results from this analysis displayed several pathways that consistently differed between both cells lines. The predicted results for the TLR7/8 cascade appeared interesting, since they showed opposite phenotypes (e.g., activation or inhibition) in each cell line. Specifically, these pathways were predicted to be inhibited in infected microglia cells (blue), but activated in infected placenta cells (orange) when comparing the three dpi and one dpi time points in ZIKV-infected cells. This pattern was repeated in all of the pathways involving TLR7/8.

### 3.3. Effect of siRNA against TLR7, TLR8, or Both

**Figure 3:**
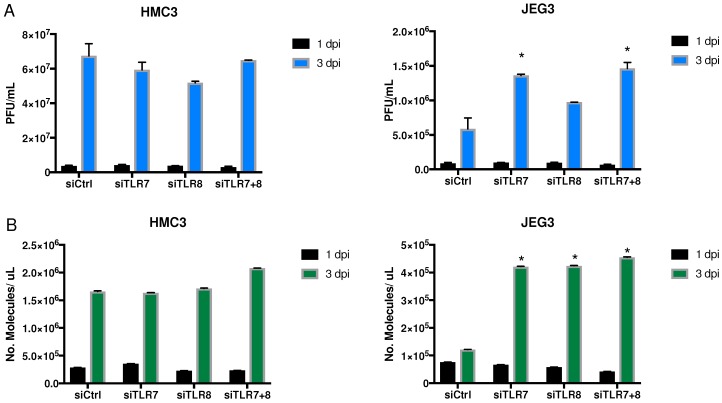
Viral and transcriptional effects of siRNAs targeting TLR7, TLR8, or TLR7 + TLR8 on ZIKV virus replication in different cell types over time. (**A**) Plaque-forming units (PFU) per mL of supernatant were measured to quantify infectious virus production at each time point in each cell type. (**B**) The number of ZIKV RNA molecules from the same samples was measured with RT-qPCR to quantify viral genome replication at both time points in each cell type (* *p* < 0.05). Error bars represent standard deviation.

**Figure 4:**
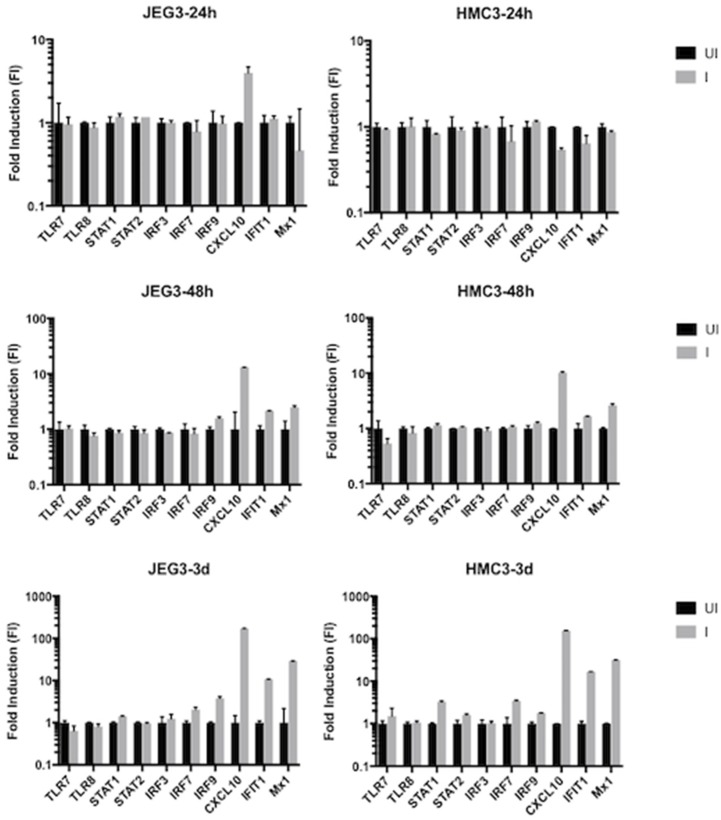
Fold induction values for genes involved in the innate immune response of time-matched mock-infected (UI) versus ZIKV-infected (I) HMC3 and JEG-3 cells at 24 h post infection (hpi), 48 hpi, and three dpi. Values were determined by calculating the fold induction (FI) using the delta-delta cycle threshold (∆∆Ct) method for each gene, normalizing the values for each gene to the UI results.

**Figure 5:**
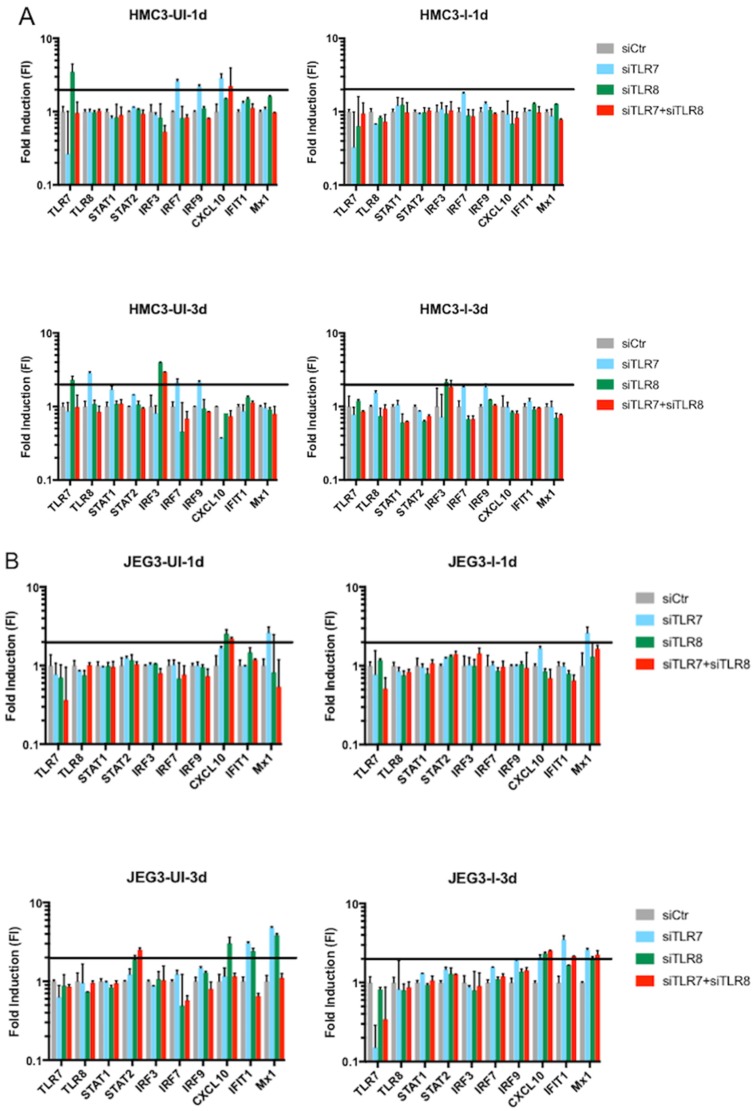
A comparison of the effects of a panel of siRNAs including TLR7, TLR8, TLR7 + TLR8, or scramble (control) on the expression of selected innate immune response factors. (**A**) HMC3 cells transfected with siRNA and either time-matched mock-infected (UI) or infected with ZIKV (I) at one dpi and three dpi. (**B**) JEG3 cells transfected with siRNA and either time-matched mock-infected (UI) or infected with ZIKV (I) at one dpi and three dpi. The horizontal black line marks the two-fold induction (FI) threshold in each plot.

We then tested the role of TLR7 and TLR8 in the host–pathogen interaction network. To do so, we used pools of siRNAs that specifically target these receptors, and measured the impact on viral replication (Figure 3) and the level of expression of a group of well-known genes involved in intracellular antiviral and innate immune responses (toll-like receptors, transcription factors, and interferon-stimulated genes) (Figure 4 and Figure 5).

#### 3.3.1. Viral Replication

We quantified viral replication by measuring the number of whole infectious viral particles released to the media through plaque assays after knocking down TLR7 and/or TLR8 (Figure 3A). We compared these numbers to the Ct values that were obtained from ZIKV RT-qPCR, and saw that they corresponded with each other (Figure 3B). We observed that siRNA against TLR7 and/or TLR8 had no effect on viral replication three dpi when compared with the siRNA control in microglia cells (HMC3). However, when the same transcripts were knocked down in placenta cells (JEG-3), ZIKV replication was significantly increased three dpi compared with the control. These assays confirmed the previous results in each of our selected cell lines.

#### 3.3.2. Innate Immune Response in Microglia and Placenta Cells

In order to determine whether other intracellular factors contributed to ZIKV replication, we quantified the level of expression of other components in the innate immune system. Specifically, we evaluated: transcription factors STAT1, STAT2, IRF3, IRF7, and IRF9; as well as the interferon-stimulated genes (ISGs) CXCL10, IFIT1, and MX1 in mock-infected (UI) and ZIKV-infected (I) placenta cells (Figure 4, left panel) and microglia cells (Figure 4, right panel). Placenta cells showed a strong induction of the ISGs at two and three days after Zika infection, but no noticeable induction of the remaining studied genes. Conversely, the microglial cells showed induction of almost the entire panel of transcripts three days after infection.

We next wanted to understand how the differential expression of these transcripts was affected after blocking TLR7, TLR8, or both (Figure 5, Supplementary Figure S2). In microglia cells (Figure 5A), after siRNA transfections, there was an initial response from some of the transcription factors at day one and three. However, that induction returned to normal when the transfection was followed by ZIKV infection. At three dpi, the ZIKV-infected microglia cells showed sporadic induction compared with uninfected cells (Figure 4), but not with cells that were ZIKV-infected and previously transfected with the specific siRNAs (Figure 5A, right lower plot). Similar results were observed in placenta cells (Figure 5B), where cells transfected with siRNAs against TLR7 and/or TLR8 showed a strong upregulation of a subset of genes (Figure 5B, left plots) that mostly disappeared when these cells were transfected and infected with ZIKV (Figure 5B, right plots). In addition, the observed upregulation of cytokines in infected placenta and microglia cells (Figure 4) was not as strong when the cells were transfected with siRNAs, or transfected and infected.

### 3.4. Role of STAT2 in Host Intracellular Response after ZIKV Infection

**Figure 6:**
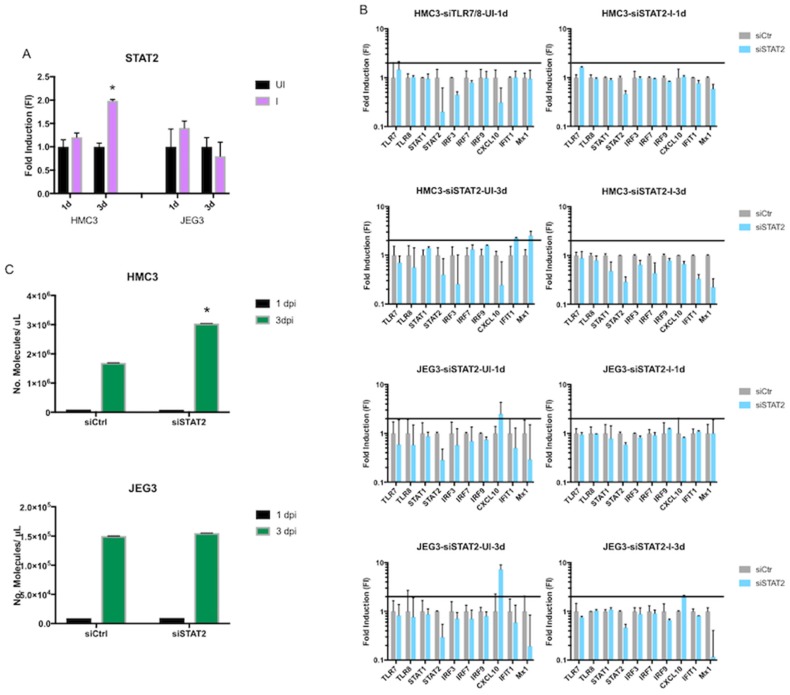
Role of STAT2 in ZIKV replication and in the intracellular response to infection. (**A**) STAT2 expression in time-matched mock-infected (UI) and ZIKV-infected (I) HMC3 and JEG-3 cells at one dpi and three dpi. (**B**) Effect of STAT2 knockdown on selected innate immune genes in time-matched mock-infected (UI) and infected (I) HMC3 and JEG-3 cells at one dpi and three dpi. The horizontal black line represents the two-fold induction (FI) threshold in each plot. (**C**) Number of ZIKV RNA molecules detected from the total RNA collected from cells treated with siRNAs against STAT2. (* *p* < 0.05).

We next decided to study the role of STAT2 in the intracellular transcriptional response after ZIKV infection. This decision was partially dependent on the results from the previous experiments performed in this study, and also based on previous reports that showed that ZIKV can degrade STAT2 in specific cells [16]. We first observed a significant upregulation of STAT2 after three days of infection in microglia cells; however, this upregulation was absent in placenta cells (Figure 6A). Transfecting cells with pools of STAT2-specific siRNAs to knockdown STAT2 transcript levels resulted in a dramatic reduction in the expression levels for genes involved in the intracellular innate immune response in both cell lines at both time points. The only exception to this trend was found for CXCL10 in placenta cells, which was upregulated after STAT2 knockdown; however, this upregulation decreased somewhat after ZIKV infection (Figure 6B). We then quantified the number of ZIKV RNA molecules in each cell type as a measure of viral replication after STAT2 knockdown. This experiment showed an increase in the ZIKV RNA molecules in microglia cells compared to the siRNA control, but no changes were observed in placenta cells (Figure 6C).

### 3.5. Role of Viral Receptor AXL in Microglia and Placenta Cells

**Figure 7:**
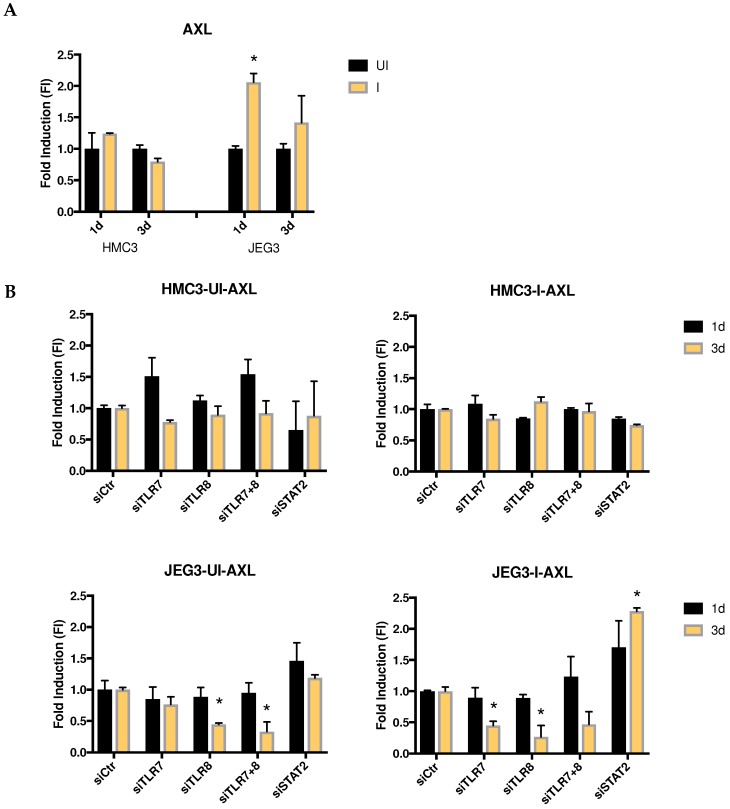
Role of AXL in ZIKV infection. (**A**) AXL fold induction ZIKV-infected (I) relative to time-matched mock-infected (UI) HMC3 and JEG-3 cells at one dpi and three dpi. (**B**) Effect of siRNAs targeting TLR7, TLR8, TLR7 + TLR8, or STAT2 on AXL expression in time-matched mock-infected (UI) and ZIKV-infected (I) HMC3 and JEG-3 cells at one dpi and three dpi. (* *p* < 0.05). Error bars represent standard deviation.

The AXL gene encodes a tyrosine protein kinase receptor that has been shown to be a cellular receptor used by the Zika virus to gain entry into host cells [23]. When we studied this receptor, AXL showed a significantly higher expression one day after infection in placenta cells when compared to uninfected cells; however, those expression levels returned to normal within three days after infection. In contrast, there was no differential-expression of AXL in infected microglia cells when compared to the uninfected cells (Figure 7A). Therefore, we wanted to test the effect of siRNAs against STAT2, TLR7, and/or TLR8 on AXL expression in these two cell lines. We observed that AXL maintained relatively normal levels of expression in microglia cells after blocking TLR7, TLR8, TLR7/8, and STAT2. However, in placenta cells, we saw that siRNA against TLR8 significantly decreased AXL expression after three days, with a similar downregulation also observed after TLR7 knockdown three days after infection. In contrast, siRNA against STAT2 in infected placenta cells significantly induced AXL expression after three days of infection when compared to control cells (Figure 7B).

## 4. Discussion

Defining the mechanisms by which ZIKV causes neuropathic effects is critical to predict future risks as well as establish control measures against the disease. In this study, we evaluated the behavior of ZIKV in two target cell lines, placenta and microglia, with the aim of understanding how the virus counteracts the intracellular antiviral response in these two cell lines during infection. Several studies have demonstrated that ZIKV infection can result in different interferon (IFN) responses depending on the cell type [24,25,26,27]. In concordance with these studies, we first demonstrated that Zika is able to replicate in both cell lines, placenta (JEG-3) and microglia (HMC3), causing cytopathic effects after four days of infection. Interestingly, the host intracellular response to ZIKV infection was different in each of the cell lines that we tested. Not only did we identify sets of significant differentially expressed genes that are involved in the intracellular innate immune response when we compared the two cell types, but we also observed differences in the activation and/or inhibition of immunological signaling pathways. Due to the different function of each cell type, we expected to observe a higher immune activation in microglia cells, which are known as the “macrophages of the brain”, to fight against any pathogen that could compromise this tissue. In contrast, the placenta cells function as a barrier to protect the fetus from any disturbance such as inflammation or immune activation that could affect its stability and development, which led us to expect an active but more subdued antiviral response.

Assuming these functional differences and based on immunological pathways analysis results, we demonstrated that knocking down the expression of TLR7 and/or TLR8 had different consequences for viral replication in each cell line. We demonstrated that TLR7/8 inhibits viral replication in placenta cells but not in microglia, as this pathway is already inhibited by the virus. Prior studies carried out with the TLR7/8 agonist R848 demonstrated that this molecule blocks ZIKV replication in monocytes and macrophages by inducing the antiviral protein Viperin [15]. Follow-up experiments will be needed to test whether Viperin is also involved in the inhibition of virus replication in placenta cells. The stronger inhibitory response after ZIKV infection on a selected group of genes when the TLR7/8 signaling pathway was blocked in both cell lines demonstrates its role in the intracellular innate immune response. It has been recently shown that the inhibition of TLR8 in ZIKV-infected trophoblasts abrogated the inflammatory cytokine responses, which is in accordance with our findings [14].

Previous investigations to determine the mechanism causing a reduction in the intracellular IFN response showed that the ZIKV NS5 protein is able to degrade STAT2, which is an IFN pathway precursor molecule [16]. However, this mechanism is not expanded to other cell types such as dendritic cells, where ZIKV is able to antagonize IFN type I response by impeding STAT1 and STAT2 phosphorylation [28]. In this study, we demonstrate that STAT2 is significantly upregulated in infected microglia cells when compared with uninfected cells, but its levels do not change during the infection of placenta cells. We validated that the virus behaves differently in each cell line to counteract the unique intracellular antiviral response, which likely reflects the functions of their respective tissues. In addition, ZIKV showed a significantly higher replication rate when STAT2 was knocked down in microglia cells compared with the control; however, no significant differences were observed in placenta cells under similar conditions. As observed when we knocked down TLR7/8, after reducing STAT2 transcript abundance, we observed a stronger inhibition of the set of genes involved in the intracellular innate immune response. These data justify performing more in-depth studies into the pathogenic mechanism(s) of Zika virus in these and other cell types that are susceptible to ZIKV infection. We expect that such experiments would greatly contribute to the discovery of specific targets for antiviral drug development.

Another controversial molecule in ZIKV infection is the cellular receptor AXL. While in some cell types it acts as a ZIKV viral entry receptor [24,29], it has been demonstrated that AXL does not always perform this role during ZIKV infection [30,31]. In accordance with these publications, our study demonstrates that AXL gene expression is different in these two cell lines. Interestingly, AXL is associated with viral infection, TLR7/8 transcript abundance, and STAT2 transcript abundance in placenta cells, since their levels vary both when these cells are infected as well as when TLR7/8 or STAT2 are knocked down. Global transcriptomic methods, such as RNAseq could be used in future experiments in order to decipher this relationship during ZIKV infection and its implications in viral pathogenesis. Such an approach would give us much more information about host–pathogen interactions and the relevant molecules and pathways involved in ZIKV pathogenesis in placenta, microglia, and/or other cells. Confirmation of these findings at the protein level will also be important to better define the mechanism of action of Zika pathogenesis and neurotropism in different cell types.

**Figure 8:**
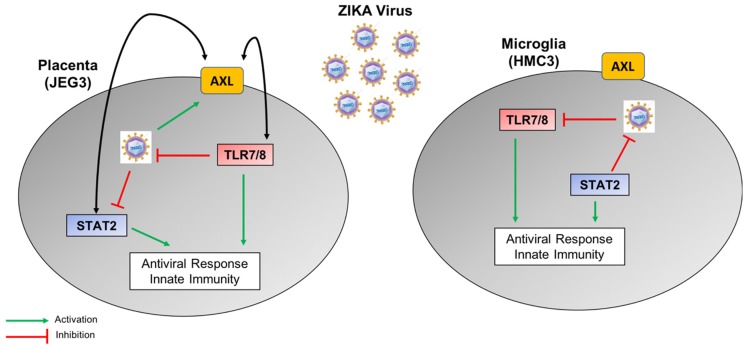
A potential schematic molecular model depicting the differing relationships between the expression of STAT2, TLR7, TLR8, and AXL during ZIKV infection in human placenta (JEG-3) and microglia (HMC3) cells.

In this manuscript, we have demonstrated that the antiviral response during ZIKV infection is highly dependent on the type of host cell being infected. Based on the results presented here, we propose a Zika virus–host cell interaction molecular model for placenta and microglia cells involving TLR7/8, STAT2, and AXL (Figure 8). This model will enable better understanding of the mechanism of action of the virus and as well as the design and development of specific antiviral drugs.

## Figures and Tables

**Table 1:** Bonferroni-corrected *p*-values of differentially affected signaling pathways detected with the signaling pathway impact analysis (SPIA) algorithm in: ZIKV-infected (I) or time-matched mock-infected (UI) cells at each of two time points in each cell type.

	HMC3 (Human Microglia)				JEG3 (Human Placenta)			
	1 dpi (I vs. UI)	3 dpi (I vs. UI)	Infected (3 dpi vs. 1 dpi)	Uninfected (3 dpi vs. 1 dpi)	1 dpi (I vs. UI)	3 dpi (I vs, UI)	Infected (3 dpi vs. 1 dpi)	Uninfected (3 dpi vs. 1 dpi)
Jak-STAT signaling pathway	7.88 × 10^−6^	6.85 × 10^−8^	2.77 × 10^−8^	6.58 × 10^−8^	7.90 × 10^−4^	1.26 × 10^−7^	1.06 × 10^−13^	2.05 × 10^−7^
NF-kappa B signaling pathway	2.05 × 10^−3^	2.13 × 10^−11^	6.23 × 10^−10^	5.13 × 10^−10^	8.00 × 10^−8^	2.12 × 10^−9^	2.16 × 10^−9^	1.02 × 10^−11^
Toll-like receptor signaling pathway	9.21 × 10^−12^	1.44 × 10^−29^	5.48 × 10^−28^	2.87 × 10^−20^	1.25 × 10^−23^	1.02 × 10^−31^	3.44 × 10^−33^	1.22 × 10^−27^
Toll-like receptor TLR1:TLR2 cascade	N.S.	1.55 × 10^−5^	1.43 × 10^−7^	1.04 × 10^−5^	4.89 × 10^−3^	9.80 × 10^−11^	1.20 × 10^−8^	7.44 × 10^−4^
Toll-like receptor TLR6:TLR2 cascade	N.S.	1.36 × 10^−5^	1.54 × 10^−7^	1.17 × 10^−5^	4.92 × 10^−3^	9.00 × 10^−11^	1.24 × 10^−8^	7.25 × 10^−4^
TRIF-mediated TLR3/TLR4 signaling	N.S.	7.07 × 10^−7^	3.30 × 10^−7^	4.47 × 10^−7^	5.53 × 10^−3^	1.11 × 10^−8^	2.12 × 10^−3^	4.70 × 10^−7^
TRAF6-mediated IRF7 activation in TLR7/**8** or 9 signaling	2.23 × 10^−2^	2.20 × 10^−3^	6.63 × 10^−9^	9.52 × 10^−6^	N.S.	N.S.	1.98 × 10^−3^	1.69 × 10^−8^
Caspase activation via extrinsic apoptotic signaling pathway	N.S.	N.S.	N.S.	2.53 × 10^−2^	8.02 × 10^−3^	2.24 × 10^−8^	4.23 × 10^−4^	2.34 × 10^−4^
IFN-alpha signaling pathway	N.S.	3.34 × 10^−3^	2.63 × 10^−3^	7.99 × 10^−3^	N.S.	1.07 × 10^−5^	2.40 × 10^−7^	N.S.
PI3K-Akt signaling pathway	N.S.	2.34 × 10^−3^	1.50 × 10^−3^	N.S.	2.86 × 10^−2^	4.47 × 10^−5^	4.66 × 10^−6^	N.S.
Toll-like receptor 7/8 (TLR7/8) cascade	N.S.	7.57 × 10^−6^	5.30 × 10^−10^	8.80 × 10^−6^	N.S.	N.S.	3.18 × 10^−2^	1.00 × 10^−5^
Toll-like receptor 9 (TLR9) cascade	N.S.	9.55 × 10^−6^	6.03 × 10^−10^	1.17 × 10^−5^	N.S.	N.S.	3.77 × 10^−2^	1.28 × 10^−5^
TRAF6-mediated induction of NFkB and MAP kinases upon TLR7/**8** or 9 activation	N.S.	5.13 × 10^−4^	5.82 × 10^−8^	1.97 × 10^−4^	N.S.	N.S.	2.74 × 10^−2^	5.23 × 10^−4^
TRAF6-mediated IRF7 activation	N.S.	4.14 × 10^−4^	2.92 × 10^−4^	3.78 × 10^−6^	N.S.	N.S.	5.97 × 10^−4^	9.19 × 10^−3^
Regulation of nuclear SMAD2/3 signaling	N.S.	7.12 × 10^−5^	1.93 × 10^−3^	N.S.	N.S.	N.S.	N.S.	4.02 × 10^−2^
TRAF3-dependent IRF activation pathway	N.S.	2.58 × 10^−3^	2.02 × 10^−3^	3.55 × 10^−3^	N.S.	N.S.	N.S.	N.S.
TRIF-mediated programmed cell death	N.S.	N.S.	N.S.	N.S.	N.S.	1.59 × 10^−8^	1.94 × 10^−3^	8.05 × 10^−4^
Apoptosis	N.S.	2.53 × 10^−2^	N.S.	3.22 × 10^−2^	N.S.	N.S.	N.S.	N.S.
Regulation of IFNA signaling	N.S.	N.S.	N.S.	N.S.	N.S.	1.85 × 10^−2^	2.23 × 10^−4^	N.S.

N.S.: Not Significant; Blue: inhibited pathway; Orange: activated pathway.

## Supplementary Materials

The following are available online at http://www.mdpi.com/1999-4915/10/11/649/s1, Figure S1: Fold-induction values of control siRNAs in untreated (UT) or positive control (PSC) samples at 1dpi and 3dpi following siRNA transfection in each HMC3 and JEG3 cells. Figure S2: Fold-induction values for each cell type at time-matched mock-infected cells after only siRNA-control transfection (UI), siRNA-control transfection plus virus infection (I), or transfected with either siRNAs targeting TLR8 or TLR7 + TLR8 (upper panel), TLR7 or TLR7 + TLR8 (middle panel) or STAT2 (lower panel) prior to infection with ZIKV at 1dpi and 3dpi in each of two human cell types. Table S1: set of primers used for measuring transcriptional expression levels through RT-PCR. Table S2: Innate and Adaptive Immune Responses RT^2^ Profiler PCR Array list of genes. Table S3: Zika virus TaqMan primers and Probe for RT-qPCR.
