# Supplementary material for: Characterizing the Different Effects of Zika Virus Infection in Placenta and Microglia Cells
Source: Viruses. 2018 Nov 18;10(11):649. doi: 10.3390/v10110649 (PMC6266000; doi:10.3390/v10110649)
Supplement: Supplementary file 1 [file viruses-10-00649-s001.zip › Supplementary_material/Supplementary table 1.pdf]

Supplementary table 1: set of primers used for measuring transcriptional expression levels through RT-qPCR.

Gene	Primer 1	Primer 2
Actin B	5′-CCTTGCACATGCCGGAG-3′	5′-ACAGAGCCTCGCCTTTG-3′
18S	5′-GGACATCTAAGGGCATCACAG-3′	5′-GAGACTCTGGCATGCTAACTAG-3′
TLR7	5′-GGAATGTAGAGGTCTGGTTGAAG-3′	5′-CCAGATATAGGATCACTCCATGC-3′
TLR8	5′-GATCCAGCACCTTCAGATGAG-3′	5′-ACTTGACCCAACTTCGATACC-3′
STAT1	5′-GATCACTCTTTGCCACACCA-3′	5′-TAGAGCATGAAATCAAGAGCCT-3′
STAT2	5′-TGAAGCGCAGTAGAAAGGTG-3′	5′-CTGAAGGATCTCTGGAATGATGG-3′
IRF3	5′-TCCGCTTCCTTCCGTGA-3′	5′-AATCCCACTCCCTTCCCA-3′
IRF7	5′-GTGGACTGAGGGCTTG-3′	5′-TCAACACCTGTGACTTCATGT-3′
IRF9	5′-CTGCCCACTCTCCACTTG-3′	5′-GTTAAGCTGAGGTCGTCTGAG-3′
CXCL10	5′-CAGTTCTAGAGAGAGGTACTCCT-3′	5′-GACATATTCTGAGCCTACAGCA-3′
IFIT1	5′-GCTCCAGACTATCCTTGACCT-3′	5′-CCACAAGACAGAATAGCCAGAT-3′
MX1	5′-GTATCACAGAGCTGTTCTCCTG-3′	5′-CTCCCACTCCCTGAAATCTG-3′
