# Supplementary material for: Characterizing the Different Effects of Zika Virus Infection in Placenta and Microglia Cells
Source: Viruses. 2018 Nov 18;10(11):649. doi: 10.3390/v10110649 (PMC6266000; doi:10.3390/v10110649)

Supplementary figure 2: Fold-induction values for each cell type at time-matched mock-infected cells after only siRNA-control transfection (UI), siRNA-control transfection plus virus infection (I), or transfected with either siRNAs targeting TLR8 or TLR7+TLR8 (upper panel), TLR7 or TLR7+TLR8 (middle panel) or STAT2 (lower panel) prior to infection with ZIKV at 1dpi and 3dpi in each of two human cell types.

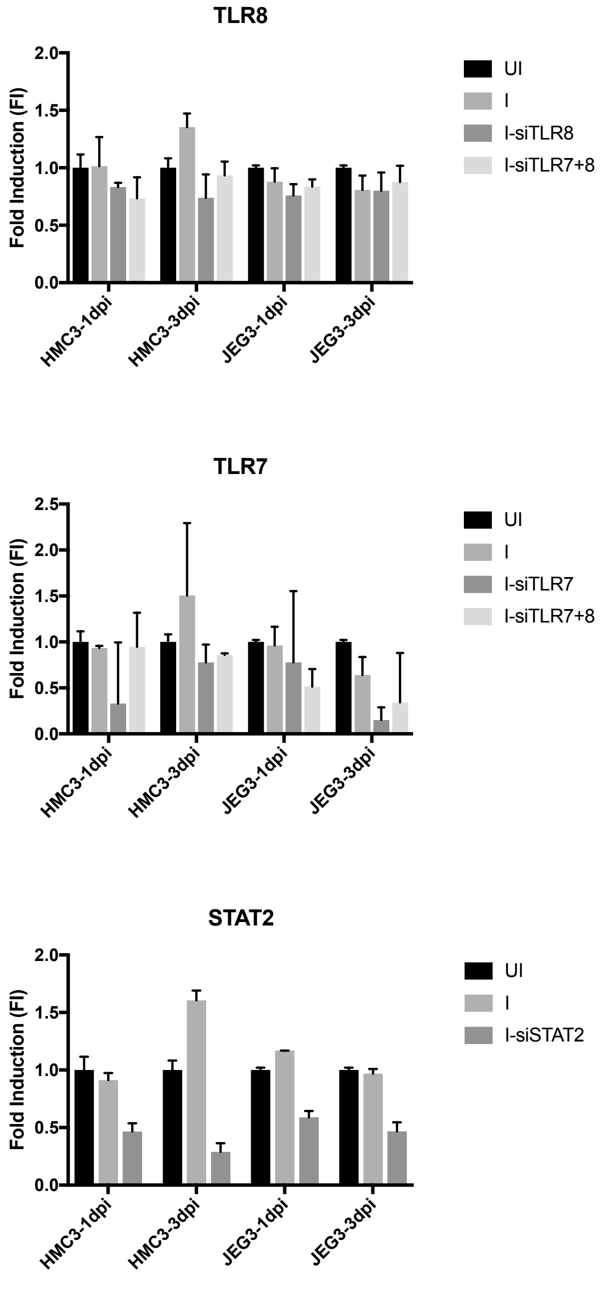

Supplement: Supplementary file 1 [file viruses-10-00649-s001.zip › Supplementary_material/Supplementary figure 2.pdf]
