# Supplementary material for: Characterizing the Different Effects of Zika Virus Infection in Placenta and Microglia Cells
Source: Viruses. 2018 Nov 18;10(11):649. doi: 10.3390/v10110649 (PMC6266000; doi:10.3390/v10110649)
Supplement: Supplementary file 1 [file viruses-10-00649-s001.zip › Supplementary_material/Supplementary table 2.pdf]

Supplementary table 2: Innate and Adaptive Immune Responses RT^2 Profiler PCR Array list of genes.

Gene ID	Gene ID	Gene ID	Gene ID	Gene ID	Gene ID
APCS	CD86	IFNAR1	IL5	MX1	TBX21
C3	CD8A	IFNB1	IL6	MYD88	TICAM1
CASP1	CRP	IFNG	CXCL8	NFKB1	TLR1
CCL2	CSF2	IFNGR1	IRAK1	NFKBIA	TLR2
CCL5	CXCL10	IL10	IRF3	NLRP3	TLR3
CCR4	CXCR3	IL13	IRF7	NOD1	TLR4
CCR5	DDX58	IL17A	ITGAM	NOD2	TLR5
CCR6	FASLG	IL18	JAK2	RAG1	TLR6
CCR8	FOXP3	IL1A	LY96	RORC	TLR7
CD14	GATA3	IL1B	LYZ	SLC11A1	TLR8
CD4	HLA-A	IL1R1	MAPK1	STAT1	TLR9
CD40	HLA-E	IL2	MAPK8	STAT3	TNF
CD40LG	ICAM1	IL23A	MBL2	STAT4	TRAF6
CD80	IFNA1	IL4	MPO	STAT6	TYK2
