# Supplementary material for: Characterizing the Different Effects of Zika Virus Infection in Placenta and Microglia Cells
Source: Viruses. 2018 Nov 18;10(11):649. doi: 10.3390/v10110649 (PMC6266000; doi:10.3390/v10110649)

Supplementary figure 1: Fold-induction values of control siRNAs in untreated (UT) or positive control (PSC) samples at 1dpi and 3dpi following siRNA transfection in each HMC3 and JEG3 cells.

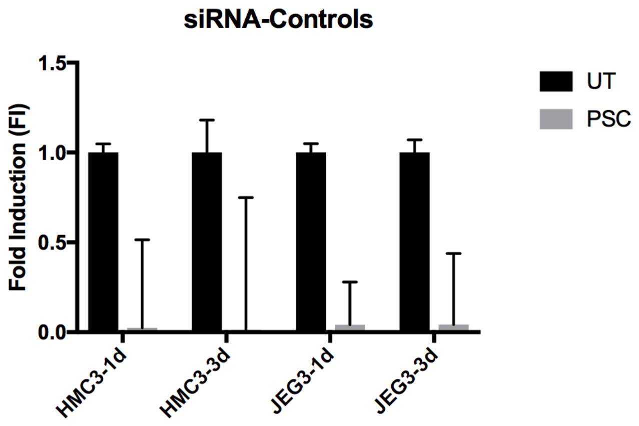

Supplement: Supplementary file 1 [file viruses-10-00649-s001.zip › Supplementary_material/Supplementary figure 1.pdf]
