# Supplementary material for: Characterizing the Different Effects of Zika Virus Infection in Placenta and Microglia Cells
Source: Viruses. 2018 Nov 18;10(11):649. doi: 10.3390/v10110649 (PMC6266000; doi:10.3390/v10110649)
Supplement: Supplementary file 1 [file viruses-10-00649-s001.zip › Supplementary_material/Supplementary table 3.pdf]

Supplementary table 3: Zika virus TaqMan primers and Probe for RT-qPCR.

**Forward primer**	5′-ATAACAGCTTTGTCGTGGATG-3′
**Reverse primer**	5′- TAACCTTGAGCCAGACACTAG-3′
**FAM-Probe**	5′- AGAGCATGGAACAGCTTTCTTGTG-3′
